# Perinatally Administered Bisphenol A as a Potential Mammary Gland Carcinogen in Rats

**DOI:** 10.1289/ehp.1306734

**Published:** 2013-07-23

**Authors:** Nicole Acevedo, Barbara Davis, Cheryl M. Schaeberle, Carlos Sonnenschein, Ana M. Soto

**Affiliations:** 1 Department of Anatomy and Cellular Biology, Tufts University School of Medicine, Boston, Massachusetts, USA; 2 Department of Pathology, Cummings School of Veterinary Medicine, Tufts University, Grafton, Massachusetts, USA

## Abstract

Background: Environmental exposure to bisphenol A (BPA) affects mammary gland development in rodents and primates. Prenatal exposure to environmentally relevant doses of BPA increased the number of intraductal hyperplasias and ductal carcinomas *in situ* by 50 days of age in Wistar-Furth rats.

Objective: We aimed to determine whether BPA exposure of dams during gestation only or throughout lactation affects the incidence of mammary gland neoplasia in female offspring.

Methods: We treated pregnant Sprague-Dawley rats with BPA at 0, 0.25, 2.5, 25, or 250 μg BPA/kg BW/day from gestational day (GD) 9 to birth and from GD9 to postnatal day (PND) 21. Mammary glands from BPA-exposed offspring were examined at four time points for preneoplastic and neoplastic lesions. To assess circulating BPA levels, we exposed pregnant rats to vehicle or 250 μg BPA/kg BW/day during gestation only or during gestation/lactation and analyzed sera from dams, fetuses, and nursing pups for total and unconjugated BPA.

Results: Total and unconjugated BPA were detected in sera from 100% of dams and fetuses and 33% of pups exposed to 250 μg BPA/kg BW/day. Unconjugated BPA levels in exposed dams and fetuses (gestational) and in exposed dams and pups (gestational/lactational) were within levels found in humans. Preneoplastic lesions developed in BPA-exposed female offspring across all doses as early as PND50. Unexpectedly, mammary gland adenocarcinomas developed in BPA-exposed offspring by PND90.

Conclusions: Our findings suggest that developmental exposure to environmentally relevant levels of BPA during gestation and lactation induces mammary gland neoplasms in the absence of any additional carcinogenic treatment. Thus, BPA may act as a complete mammary gland carcinogen.

Citation: Acevedo N, Davis B, Schaeberle CM, Sonnenschein C, Soto AM. 2013. Perinatally administered bisphenol A acts as a mammary gland carcinogen in rats. Environ Health Perspect 121:1040–1046; http://dx.doi.org/10.1289/ehp.1306734

## Introduction

Cumulative exposure to ovarian steroids during a woman’s lifetime represents the most well-defined risk factor for the development of breast cancer. Epidemiological studies have suggested that increased estrogen levels in the fetal environment are associated with an increased risk of breast cancer during adult life (Braun et al. 1995; Ekbom et al. 1992; Potischman and Troisi 1999). The synthetic estrogen diethylstilbestrol (DES), prescribed from the 1940s to the 1970s to prevent miscarriage, is recognized as a seminal example of a human transplacental carcinogen for the multitude of adverse effects manifested in adult offspring. The effects of DES include increased risk of vaginal clear cell carcinoma, reproductive tract malformations, poor pregnancy outcomes, and compromised immune systems (Herbst et al. 1971; Hoover et al. 2011), as well as increased risk of developing breast cancer after 40 years of age (Hoover et al. 2011; Troisi et al. 2007). Another synthetic estrogen, bisphenol A (BPA), is currently one of the highest volume chemicals produced worldwide, with a global production capacity of 11.5 billion pounds in 2008 (Burridge 2008; vom Saal et al. 2007). BPA is used in the production of polycarbonate plastics, epoxy resins, dental sealants and composites, and thermal receipt paper. Incomplete polymerization of BPA leads to leaching of the chemical and subsequent human exposure, as evidenced by the detection of BPA in human urine, serum, maternal and fetal plasma, amniotic fluid, placenta, and adipose tissue (Calafat et al. 2005; Fernandez et al. 2007; Ikezuki et al. 2002; Schönfelder et al. 2002; Vandenberg et al. 2007a; Zalko et al. 2011). Although oral exposure through ingestion of food and beverages was considered the main route in humans, recent studies have indicated that humans are also exposed to BPA through inhalation as well as via absorption through the skin and mucosal membranes of the mouth, and that these routes are not negligible (Vandenberg et al. 2013). The U.S. Environmental Protection Agency (EPA) has calculated an oral reference dose for BPA of 50 μg BPA/kg body weight (BW)/​day based on a lowest observed adverse effect level of 50 mg/kg BW/day (U.S. EPA 1993; Welshons et al. 2003). A review of more than two dozen biomonitoring studies that used analytical chemistry methods to measure BPA in healthy adults reported the detection of mean unconjugated BPA levels in the range of 1 ng/mL in blood (Vandenberg et al. 2010). The distinction between unconjugated and conjugated BPA is especially important in blood because the unconjugated form is considered the active form and has estrogenic activity (Thomas and Dong 2006; Watson et al. 2005). Recent pharmacokinetic analyses in nonhuman primates suggested that daily oral exposure to 400 μg BPA/kg BW/day is sufficient to produce serum concentrations of unconjugated BPA in the range measured in humans (Taylor et al. 2011) and that prenatal exposure to this dose altered the developing mammary glands of female rhesus monkeys (Tharp et al. 2012).

Several studies have reported that fetal exposure to low doses of BPA altered the development of the rodent mammary gland, which manifested from the time of exposure and was exacerbated at puberty and beyond (Markey et al. 2001; Munoz de Toro et al. 2005; Vandenberg et al. 2007b, 2008). Perinatal exposure to BPA increased estrogen and progesterone sensitivity in the mouse mammary gland (Ayyanan et al. 2011; Wadia et al. 2007). Together, these results suggest that perinatal exposure to BPA may increase the propensity to breast carcinogenesis. In a study supporting this assessment, rats exposed prenatally to 2.5 μg BPA/kg BW/day showed a significant increase in the number of mammary gland intraductal hyperplasias at postnatal day (PND) 50 and PND90 compared with controls; exposure to 250 or 1,000 μg BPA/kg BW/day resulted in the development of ductal carcinomas *in situ* (DCIS) (Murray et al. 2007). Durando et al. (2007) reported that rats exposed prenatally to 25 μg BPA/kg BW/day displayed a higher number of ductal hyperplasias associated with desmoplasia in adulthood. These authors also found that nitrosomethylurea administered at 50 days of age at doses that fail to induce tumors in control animals elicited the development of mammary carcinomas in females perinatally exposed to BPA. In rats or mice exposed perinatally to BPA, administration of the chemical carcinogen dimethylbenzanthracene (DMBA) during adulthood resulted in increased tumor incidence and decreased tumor latency compared with animals exposed to DMBA alone (Jenkins et al. 2009; Weber Lozada and Keri 2011); BPA also shifted the window of susceptibility to DMBA (Betancourt et al. 2010). In addition, lactational exposure to BPA between PND2 and PND20 increased tumor incidence in conjunction with a DMBA challenge (Jenkins et al. 2009).

As a follow-up to our previous work in Wistar-Furth rats (Murray et al. 2007), we examined the effect of duration of BPA exposure over a wide range of concentrations on the induction of preneoplastic lesions and DCIS in Sprague-Dawley rats, a strain used extensively for toxicology and carcinogenesis studies by the National Toxicology Program. To relate this evidence to human biomonitoring data, we measured the internal levels of BPA in serum of these rats. Unexpectedly, at scheduled sacrifice times, we observed large mammary carcinomas (> 1 cm^2^ in diameter) occurring at internal doses relevant to human exposure, suggesting that BPA may act as a complete carcinogen.

## Materials and Methods

*Animals*. Sexually mature virgin female Sprague-Dawley rats (8–10 weeks of age; Taconic, Germantown, NY) were maintained in temperature- and light-controlled (14 hr/10 hr light/dark) conditions in the Tufts University School of Medicine Division of Laboratory Animal Medicine. Experimental procedures were approved by the Tufts University–Tufts Medical Center Animal Research Committee, and all animals were treated humanely and with regard for alleviation of pain in accordance with the *Guide for the Care and Use of Laboratory Animals* (Institute of Laboratory Animal Resources, National Research Council 2011). Cages, water bottles, and bedding tested negligible for estrogenicity by the E-SCREEN assay (Soto et al. 1992). Food (Harlan Teklad 2018 Rodent Diet, Harlan Teklad, Indianapolis, IN) was supplied *ad libitum*. Estrogenicity of the feed was measured at 8–15 fmol of estrogen equivalents per gram, a negligible amount (Soto et al. 1992). Female rats were mated with Sprague-Dawley males. The morning on which sperm was observed in vaginal smears was designated gestational day (GD) 1.

*Fetal and neonatal exposure to BPA.* To explore its role on neoplastic development, we administered BPA subcutaneously via Alzet osmotic pumps (Durect Corp., Cupertino, CA), with the dose calculated based on the weight of the dam at day 7 of pregnancy. Dams (*n* = 9–12/dose/exposure period) were implanted with pumps on day 9 of pregnancy to administer vehicle (50% dimethyl sulfoxide; Sigma Chemical Co., St. Louis, MO) or 0.25, 2.5, 25, or 250 μg BPA/kg BW/day. For convenience, these doses are subsequently referred to as BPA0.25, BPA2.5, BPA25, and BPA250, respectively.

We examined two different exposure periods. For animals exposed only through gestation, dams were implanted with pumps (catalog no. 2002; Durect Corp.) designed to deliver continuously up to 14 days (see Supplemental Material, Figure S1A). Pumps were implanted after 24 hr equilibration, according to the manufacturer’s specifications. For animals exposed through gestation and lactation, dams were implanted with pumps (catalog no. 2004; Durect Corp) designed to deliver continuously up to 28 days (see Supplemental Material, Figure S1B). These pumps were implanted after 48 hr of equilibration. In both groups, animals delivered normally and litters were culled to 10 individuals on PND2. We distributed all female offspring (*n* = 9–12/dose/​age at sacrifice per exposure group) so that each litter was represented only once. We harvested mammary gland tissue at PND50, PND90, PND140, and PND200. The fourth left inguinal mammary gland was fixed and processed for paraffin embedding, and the contralateral gland was whole mounted and stained with carmine as previously described (Murray et al. 2007).

*Histological and whole mount analysis.* Three 5-µm sections separated by 50 µm were used to assess the presence of preneoplastic and neoplastic lesions in mammary glands of PND50 females, with five animals sampled per dose per exposure group. We visualized histological sections with an Axioskop 2 Plus microscope and captured images with an AxioCAm HR color digital camera and Axiovision software, version 4.5 (all from Carl Zeiss, München-Hallbergmoos, Germany). We assessed the incidence of total ductal hyperplasia as previously described (Murray et al. 2007). Briefly, the leading edge and terminal end buds (TEBs) were localized; a 4-mm^2^ box was drawn starting 400 μm from the most proximal TEB; and all of the ducts within this area were counted. Usual intraductal hyperplasia (UDH) was characterized by an increase in monomorphic ductular epithelial cell layers (≥ 3 cells thick) typified by cuboidal or columnar pseudostratified epithelial cells that maintained a perpendicular orientation around the basement membrane. Preneoplastic lesions (atypical ductal hyperplasia; ADH) were characterized by an increase in monomorphic to mildly pleomorphic ductular epithelial cell layers, typified by flattened cuboidal epithelial cells that more intensely exhibited eosinophilic cytoplasmic staining and/or slightly enlarged hyperchromatic or vesicular nuclei. Neoplastic lesions (DCIS) were distinguished from ADH by increased layers of disorganized, pleomorphic ductular epithelial cells bridging and occluding the lumen but maintaining an intact basement membrane (Davis and Fenton 2013).

Whole mounts of mammary glands harvested at PND50, PND90, PND140, and PND200 (*n* = 9–12/treatment/age/exposure group) were assessed for proliferative lesions. Whole mounts were viewed with a Stemi 2000 stereomicroscope (Carl Zeiss). Lesions (< 10 mm in diameter) and tumors (≥ 10 mm in diameter) were excised, sectioned, and stained with hematoxylin and eosin (H&E) for diagnosis.

*Quantification of circulating levels of BPA.* We conducted a pilot experiment to determine the levels of detectability of BPA in the serum of pregnant dams. Based on dam weight at GD7, pregnant rats (*n* = 6/​dose) were implanted with pumps on GD9 to deliver vehicle, BPA25, or BPA250, and blood was collected by cardiac puncture 72 hr later. Samples were frozen and shipped to the Centers for Disease Control and Prevention, where serum samples were analyzed for total and unconjugated BPA as described by Ye et al. (2008). Briefly, serum was either treated with β-glucuronidase/sulfatase to estimate the concentration of total BPA (conjugated plus unconjugated), or processed without enzymatic treatment to estimate the concentration of unconjugated BPA. Then, serum concentrations were quantified using on-line solid phase extraction coupled to high performance liquid chromatography–isotope dilution tandem mass spectrometry. The limit of detection (LOD), the lowest amount of an analyte that can be detected with a defined probability, was 0.3 ng/mL. The limit of quantification (LOQ), calculated as three times the LOD, defines the point at which data attain statistical significance.

In pregnant dams, total serum BPA was undetectable in all vehicle-treated dams (*n* = 6) and detectable—although below the LOQ of 0.9 ng/mL—in 50% of dams treated with BPA25 (mean ± SD, 0.37 ± 0.27 ng/​mL; *n* = 6). Unconjugated BPA was detected in 100% of dams (*n* = 6) treated with BPA250 (total BPA, 3.45 ± 2.63 ng/mL; unconjugated BPA, 0.83 ± 0.31 ng/mL).

On the basis of these results, we conducted a second experiment using either vehicle or BPA250 and divided the pregnant animals into two groups. One group (corresponding to the gestational-only exposure group) was designed to measure fetal exposure near the end of gestation to ensure the collection of a sufficient volume of fetal serum. Dams were weighed at GD17 to calculate the BPA dose to be administered, and animals were implanted at GD18 with pumps (catalog no. 2002). On GD21 animals were killed, serum was collected from each dam and from fetuses; fetal sera from each litter were pooled (see Supplemental Material, Figure S1C).

The second group (corresponding to the gestational/lactational exposure group) was designed to measure BPA levels in dams and pups at PND10. Dams were weighed at GD7 and implanted with pumps (catalog no. 2004) on GD9. Animals delivered normally and litters were culled to 10 on PND2. Dams and pups were killed on PND10 and the serum collected (see Supplemental Material, Figure S1D); serum from the pups was pooled by litter. Serum samples were analyzed for total and unconjugated serum BPA at the Centers for Disease Control and Prevention as described above.

*Statistical analyses.* All calculated parameters and statistical significance were determined using SPSS statistical software (IBM SPSS Statistics 20; IBM, Chicago, IL). For the statistical calculations involving total and unconjugated BPA concentrations, we used the instrument-generated values, even if they were below the LOD, to run Student’s *t*-tests. All results are presented as mean ± SD. Overall differences in preneoplastic lesions were analyzed by analysis of variance (ANOVA), and differences in tumor incidence were analyzed via the chi-square test. For all statistical tests, results were considered significant at *p* < 0.05.

## Results

*BPA in serum of dams and their offspring following continuous exposure to BPA.* In the serum of dams, fetuses, and pups, BPA was present mainly in its conjugated form. Total and unconjugated BPA serum concentrations were detectable in 100% of pregnant dams and fetuses exposed gestationally to BPA250. The mean serum concentration of total BPA was significantly higher in exposed fetuses compared with their vehicle controls, and the difference between mean unconjugated BPA levels between exposed fetuses and their controls approached significance (*p* = 0.051; Table 1). Unconjugated BPA was significantly higher in exposed dams compared with controls. The average total BPA in the serum of BPA-exposed fetuses was four-times greater than in the serum of the dams (*p* = 0.004). In the group exposed gestationally/lactationally, total and unconjugated serum BPA were detectable in 100% of BPA-treated lactating dams, but total BPA was detectable in only 33% of the BPA-exposed pups (Table 2). Serum concentrations of total and unconjugated BPA were significantly higher in exposed lactating dams than in controls (Table 2). Total serum BPA measured in exposed pups was significantly higher than in controls; however, all values were below the LOQ. The mean total BPA concentration in the exposed pups (0.38 ng/mL) was significantly lower than in their respective dams (16.50 ng/mL; *p* = 0.03). Because BPA is ubiquitously present in the environment, extensive measures were taken throughout the process, from blood sampling to the final assay, to reduce contamination. Detection of predominantly conjugated versus unconjugated BPA in the serum of dams, fetuses, and pups suggests that external contamination, if it occurred, was not systematic or extensive.

**Table 1:** Circulating levels (internal dose) of BPA measured in serum from dams and their fetuses at GD21 following continuous exposure during gestation only (GD18–GD21).

BPA dose (μg/kg BW/day)	Animals	Total BPA			Unconjugated BPA			T:U BPA ratio
		Incidence of detectable BPA	Mean ng/mL)	Range (ng/mL)	Incidence of detectable BPA	Mean (ng/mL)	Range (ng/mL)	
0	Dams	3/6^*a*^	< LOD	0.0–0.4^*b*^	0/6	< LOD	0.0–0.2^*b*^	NA
0	Fetuses	0/5	< LOD	0.1–0.2^*b*^	0/5	< LOD	0.0–0.05^*b*^	NA
250	Dams	4/4	6.13 ± 3.88	2.5–11.4	4/4	1.25 ± 0.10*	1.2–1.4	5:1
250	Fetuses	4/4	27.90 ± 8.96*	16.7–36.7	4/4	0.63 ± 0.39	0.4–1.7^*c*^	44:1
Abbreviations: NA, not applicable; T:U, total BPA:unconjugated BPA. Values are expressed as mean ± SD; *n *= 4–6 animals/treatment group. ^***a***^Number of animals with BPA measurements at or above the LOD (0.3 ng/mL; the lowest amount of BPA that can be detected with a defined probability). ^***b***^All concentrations were at or below the LOQ (0.9 ng/mL; the lowest amount of BPA that can be quantitatively determined with suitable precision and accuracy). ^***c***^One or more concentrations were at or below the LOQ (0.9 ng/mL). *p < 0.05 compared with vehicle control by Student’s *t*-test.								

**Table 2:** Circulating levels (internal dose) of BPA measured in serum from lactating dams and their nursing pups at PND10 following continuous exposure during gestation/lactation (GD9–PND10).

BPA dose (μg/kg BW/day)	Animals	Total BPA			Unconjugated BPA			T:U BPA ratio
		Incidence of detectable BPA	Mean (ng/mL)	Range (ng/mL)	Incidence of detectable BPA	Mean (ng/mL)	Range (ng/mL)	
0	Dams	2/6^*a*^	0.35 ± 0.43	0–0.9^*b*^	0/6	< LOD	0.0–0.3^*b*^	NA
0	Pups	0/6	< LOD	0–0.1^*b*^	0/6	< LOD	NA	NA
250	Dams	6/6	16.50 ± 13.00*	6–38.8	6/6	1.25 ± 0.44*	0.9–2.1^*c*^	13:1
250	Pups	2/6^*a*^	0.38 ± 0.26*	0.2–0.8^*b*^	0/6	< LOD	0.0–0.1^*b*^	NA
Abbreviations: NA, not applicable; T:U, total BPA:unconjugated BPA. Values are expressed as mean ± SD; *n *= 6 animals/treatment group. ^***a***^Number of animals with BPA measurements at or above the LOD (0.3 ng/mL; the lowest amount of BPA that can be detected with a defined probability). ^***b***^All concentrations were at or below the LOQ (0.9 ng/mL; the lowest amount of BPA that can be quantitatively determined with suitable precision and accuracy). ^***c***^One or more concentrations were at or below the LOQ (0.9 ng/mL). *p < 0.05 compared with vehicle control by Student’s *t*-test.								

**Figure 1:**
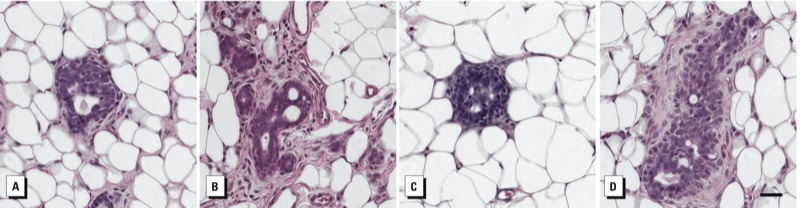
Representative photomicrographs of preneoplastic and neoplastic lesions observed in PND50 mammary glands from BPA-exposed rats. (*A*) UDH (usual intra­ductal hyperplasia) from rat after gestational/lactational exposure to BPA25. (*B,C*) ADH (atypical ductal hyperplasia) from rats after gestational/lactational exposure to BPA25 and BPA2.5, respectively. (*D*) DCIS from rat after gestational/lactational exposure to BPA25. Bar = 50 μm.

*Preneoplastic and neoplastic lesions developed in perinatally exposed mammary glands.* We analyzed histological sections at PND50 for incidence of preneoplastic and neoplastic lesions (Figure 1). The incidence of UDH was not significantly different between the BPA and vehicle controls for either exposure period (data not shown). However, histological assessment of glands for ADH or DCIS showed that the incidence of ADH among glands from females exposed to all doses of BPA during both exposure periods ranged from 0 to 60% compared with no incidence (0%) in the vehicle controls (Table 3). We diagnosed DCIS in a gland from one female rat exposed gestationally/lactationally to BPA25 (Figure 1D).

**Table 3:** Preneoplastic and neoplastic lesions observed at PND50 in histological sections of mammary glands of BPA-exposed females.

BPA exposure (μg/kg BW/day)	Gestational exposure		Gestational/lactational exposure	
	Incidence [*n* (%)]	Diagnosis	Incidence [*n* (%)]	Diagnosis
0	0/5 (0)	NA	0/5 (0)	NA
0.25	3/5 (60)	ADH	0/5 (0)	NA
2.5	1/5 (20)	ADH	1/5 (20)	ADH
25	0/5 (0)	NA	1/5 (20)	DCIS, ADH
250	2/5 (40)	ADH	1/6 (17)	ADH
Abbreviations: ADH, atypical ductal hyperplasia; DCIS, ductal carcinoma *in situ; *NA, not applicable (no lesion detected for diagnosis). Differences in observed lesions following treatment were analyzed by ANOVA and found not to be significant.				

**Figure 2:**
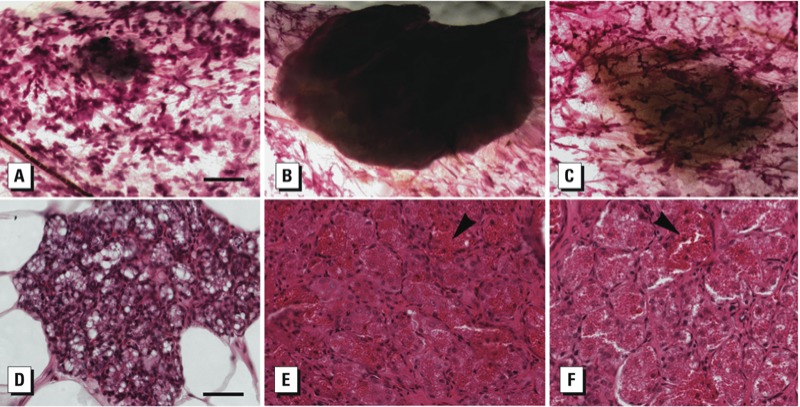
Representative photomicrographs of presumptive lesions detected in mammary gland whole mounts from PND90 and PND140 females. (*A*) Lesion (diameter, 1.7 mm) from rat at PND90 after gestational exposure to BPA250. (*B*) Lesion (diameter, 6.3 mm) from rat at PND90 after gestational/lactational exposure to BPA25. (*C*) Lesion (diameter, 5.8 mm) from rat at PND140 after gestational/lactational exposure to BPA0.25. Lesions were excised, sectioned, and H&E stained for diagnosis; bar = 1 mm. (*D,E,F*) Representative histological sections of (*A*), (*B*), and (*C*), respectively. Lobular alveolar hyperplasia was characterized by infiltration of mammary fat pad with glandular acini (*D*) and/or by focal, irregular proliferation of alveolar epithelium (*E,F*) with secretory activity (arrowheads). Bar = 50 μm.

We observed mammary gland lesions in whole mounts by PND90 (Figure 2). Four animals exhibited proliferative lesions in their mammary glands at PND90 and PND140 following either gestational-only or gestational/lactational exposure (Table 4): one lesion, diagnosed as a benign microfibroadenoma, in a mammary gland from a vehicle-exposed animal at PND140, and three lesions that were diagnosed as lobular alveolar hyperplasia in three different females, one each exposed to BPA0.25, BPA25, or BPA250.

**Table 4:** Proliferative mammary gland lesions and tumors observed at PND90, PND140, and PND200 following either gestational only or gestational/lactational exposure to BPA.

Exposure group/BPA dose (μg/kg BW/day)	Incidence		Diagnosis (age)
	Total lesions	Cancer	
Gestational
0	1/35	0/35	Microfibroadenoma^*a*^ (PND140)
0.25	1/31	1/31	Adenocarcinoma^*b,c*^ (PND200)
2.5	1/28	1/28	Adenocarcinoma^*b,c*^ (PND90)
25	0/23	0/23	NA
250	2/30	1/30	Lobular alveolar hyperplasia^*a*^ (PND90), adenocarcinoma^*b,c*^ (PND140)
Gestational/lactational
0	0/30	0/30	NA
0.25	1/27	0/27	Lobular alveolar hyperplasia^*a*^ (PND140)
2.5	2/30	1/30	Adenocarcinoma^*b,c*^ (PND140), fibroadenoma^*a,c*^ (PND200)
25	2/28	1/28	Lobular alveolar hyperplasia^*a*^ (PND90), adenocarcinoma^*b,c*^ (PND140)
250	0/33	0/33	NA
NA, not applicable (no lesion or tumor detected for diagnosis). Ratios indicate the number of animals with the lesion or tumor relative to the total number of animals per dose per exposure period for PND90, PND140, and PND200 combined. Differences in tumor incidence following treatment were compared with vehicle by chi-square test and found not to be significant. ^***a***^Benign. ^***b***^Malignant. ^***c***^Tumor.			

**Figure 3:**
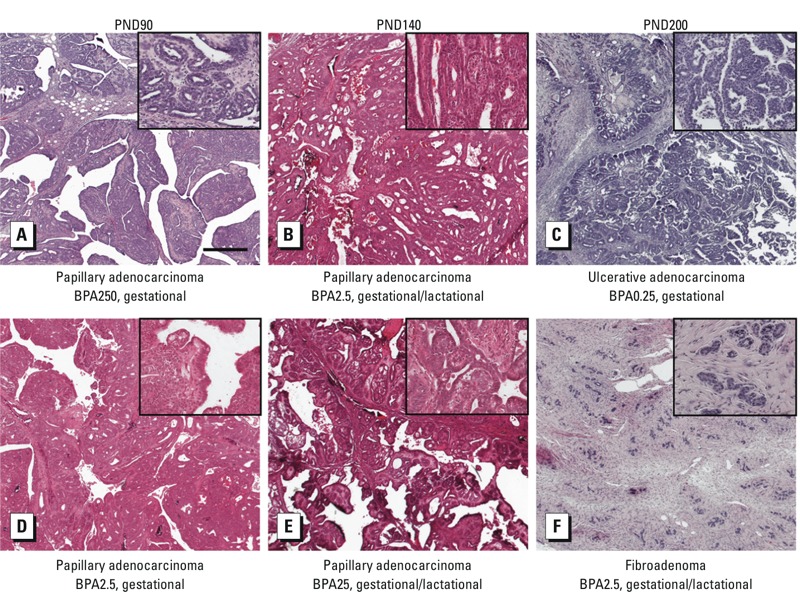
Photomicrographs of H&E-stained tumors from BPA-treated rats at time of sacrifice at PND90 (*A,D*), 140 (*B,E*), and 200 (*C,F*) in gestationally and gestationally/lactationally exposed females. (*A,B,D,E*) Papillary adenocarcinoma from rats exposed gestationally to BPA250 (*A*) or BPA2.5 (*D*), or gestationallly/lactationally to BPA2.5 (*B*) or BPA25 (*E*). (*C*) Ulcerative adenocarcinoma from rat exposed gestationally to BPA0.25. (*F*) Fibroadenoma from rat exposed gestationally/lactationally to BPA2.5. Bar = 500 μm. Insets: Magnification at 10× base image to show detail of tumor morphology.

*Malignant tumors developed following perinatal exposure to BPA.* We detected tumors at PND90, PND140, and PND200 in animals exposed to BPA across all doses and exposure times. A total of six mammary gland tumors were observed in females exposed perinatally to BPA at doses ranging from BPA0.25 to BPA250 (*n* = 230; Table 4). Five tumors were diagnosed histopathologically as adenocarcinomas and one was diagnosed as a benign fibroadenoma (Figure 3). No malignant tumors were detected in any vehicle-treated control animals (*n* = 65).

## Discussion

Developmental exposure to BPA has been shown to increase the propensity to mammary gland carcinogenesis in rodents (Betancourt et al. 2010; Durando et al. 2007; Jenkins et al. 2009; Murray et al. 2007; Weber Lozada and Keri 2011). In the present study, we observed that perinatal exposure to human-relevant internal doses of BPA—in the absence of additional exposure to chemical carcinogens—was associated with the induction of malignant mammary gland tumors and other lesions in adult female rats. To correlate dose and effect, we assessed the internal BPA dose in exposed dams and their offspring. In the BPA250 gestational exposure group, we detected significantly higher levels of unconjugated BPA in both dams and fetuses than in controls; these high levels were within the range of the levels detected in human serum (Table 1). This validates the use of osmotic pumps as an effective route of BPA administration to the fetus. Remarkably, at GD21 following gestational exposure to BPA, the average total BPA measured in fetuses was four times greater than that measured in dams. Although UDP-glucuronosyltransferase-2B1—the major liver enzyme responsible for conjugation (inactivation) of BPA via glucuronidation—shows little to no activity in the fetal rat liver (Yokota et al. 1999), the concentrations of glucuronidated BPA in the placenta and fetus have been shown to be higher than in maternal blood following administration of BPA to dams (Takahashi and Oishi 2000; Zalko et al. 2003).

Total and unconjugated BPA concentrations measured in lactating dams at PND10 following gestational/lactational BPA exposure were higher than in their nursing pups (Table 2). In fact, unconjugated BPA was undetectable in all the pups examined, whereas total BPA was detected in 33% of the BPA-exposed pups. These results suggest that when a dam is continuously exposed to a constant dose of BPA during gestation and lactation, the neonate is exposed to lower levels of BPA than the fetus, probably because of the lack of transfer in milk. Doerge et al. (2010) reported comparable results, with serum concentrations of unconjugated BPA undetectable and total BPA approximately 300 times lower in suckling rat pups than in their dams; these authors attributed the low plasma levels observed in pups to low BPA intake from the mother’s milk. Applying this relationship to our internal dose study, one may conclude that the pups from the BPA250 dams were lactationally exposed to 0.8 μg total BPA/kg BW/day, below the current U.S. EPA reference dose (U.S. EPA 1993). The increase in BPA levels in the dams at PND10 and the significant decrease in BPA in the pups compared with fetuses could also be attributed to maternal grooming practices, as suggested for other chemicals (White et al. 2007).

Oral administration of tritiated BPA has been shown to result in a linear relationship between the administered dose and unconjugated serum BPA concentrations in both rodents and primates (Taylor et al. 2011). In the present study, the mean unconjugated serum BPA concentration in dams exposed either gestationally or gestationally/lactationally to BPA250 was 1.25 ng/mL, even though the mean total BPA concentrations were almost three times higher in dams exposed gestationally/lactationally than only gestationally. Assuming linearity of circulating levels with dose, the concentrations of unconjugated BPA following exposure to BPA0.25, BPA 2.5, and BPA25 could be estimated around 0.00125, 0.0125, and 0.125 ng/mL, respectively, levels below the LOD of our method (0.3 ng/mL). However, at these undetectable levels, we have clearly seen effects in the mammary gland, both in the present data set and in our previous study (Murray et al. 2007).

Earlier studies provided evidence that perinatal exposure to low doses of BPA resulted in altered morphogenesis of the rodent mammary gland that first manifests during the exposure period. As the gland undergoes further changes under the influence of ovarian and pituitary hormones, morphological alterations become more pronounced after the onset of puberty and throughout adulthood (Munoz de Toro et al. 2005; Vandenberg et al. 2007b). In the present study, nonneoplastic lesions diagnosed as lobular alveolar hyperplasia were observed in whole mounts of mammary glands at PND90 and PND140. These lesions have been associated with the administration of xenobiotics that act as estrogen receptor (ER) agonists (Biegel et al. 1998) or dopaminergic receptor antagonists that cause prolactinemia in female rats (Lotz and Krause 1978; Lucas et al. 2007). A previous study showed that a single injection of the carcinogen *N*-nitrosomethylurea to postpubertal virgin female rats resulted in secretion of α-lactalbumin from lobular alveolar structures in the PND200 mammary gland (Murray et al. 2009). Therefore, the increased secretory material in the ducts and/or alveoli in the lesions observed in the present study may be due to xenobiotic-induced prolactinemia.

In this study, we examined whether exposure to environmentally relevant doses of BPA influenced the development of preneoplastic and neoplastic lesions in Sprague-Dawley rats. Unlike our previous study in Wistar-Furth rats (Murray et al. 2007), we observed no difference in the incidence of UDH in Sprague-Dawley females exposed to BPA compared with vehicle controls. This could be attributed to strain differences in the overall histoarchitecture of the mammary gland in the peripubertal phase (Fenton S, personal communication). Alternatively, the time course of development and regression of these lesions may be different in these strains. In the present study, both ADH and DCIS were identified only in glands of females exposed to BPA, at doses as low as BPA0.25.

To our knowledge, the induction of malignant tumor formation following developmental exposure to environmentally relevant levels of BPA has not been previously reported. As an unexpected outcome of the present study, adenocarcinomas were identified in females as early as PND90 and at doses as low as BPA0.25. It is important to note that no vehicle-exposed control animals developed malignant tumors throughout the duration of the study. Historical data on the natural occurrence of neoplastic lesions in control female Sprague-Dawley rats from large carcinogenicity trials established that there is evidence that spontaneous malignant tumors do not occur before PND210, and in fact most occurred after PND350 (Ikezaki et al. 2011; National Toxicology Program 2007, 2010; Son and Gopinath 2004). Although the incidence of tumor development was not statistically significant, the highly adverse nature of carcinomas validates the biological importance of reporting this outcome, as this information warrants further studies.

How BPA contributes to the initiation and progression of neoplasia is still unknown; however, perinatal exposure to low doses of BPA has been shown to affect the hypothalamic–​pituitary–ovarian axis via *a*) altered development of the hypothalamic nuclei essential for cyclic gonadotropin release (Rubin et al. 2006), *b*) disrupted estrous cyclicity of exposed individuals (Rubin et al. 2001), and *c*) increased sensitivity of the adult mammary gland to ovarian hormones (Ayyanan et al. 2011; Munoz de Toro et al. 2005). It is likely that BPA, like DES, may induce carcinogenesis by acting as an estrogen. During fetal life, ERs α and β are present only in stromal cells; epithelial ER expression begins at the end of gestation (Vandenberg et al. 2007b). Wadia et al. (2013) reported that low-dose BPA exposure (250 ng/kg BW/​day) during mouse fetal development altered the composition and organization of the extracellular matrix and accelerated maturation of the presumptive fat pad, an event necessary for ductal invasion and branching. This suggests that BPA acts directly on the stroma, which may in turn alter the development of the epithelium, as evidenced by increased ductal area and delayed lumen formation during the period of exposure (Vandenberg et al. 2007b). These data are compatible with the tissue organization field theory of carcinogenesis that posits that carcinogens alter the reciprocal interactions between stroma and epithelium (Soto and Sonnenschein 2011), as shown by tissue recombination studies (Maffini et al. 2004).

According to the U.S. EPA, a carcinogen is a “chemical or physical agent capable of causing cancer” (U.S. EPA 2005), a definition that does not specify the mechanism(s) by which the cancer is induced; it only identifies the consequence of an insult. Thus, by this definition, and based on the data from the present study, BPA may act as a complete mammary gland carcinogen.

## Correction

The title and two summary statements were revised from the Advance Publication version of the manuscript to clarify the conclusions of the study.

## Supplemental Material

(430 KB) PDF

## References

[r1] Ayyanan A, Laribi O, Schuepbach-Mallepell S, Schrick C, Gutierrez M, Tanos T (2011). Perinatal exposure to bisphenol A increases adult mammary gland progesterone response and cell number.. Mol Endocrinol.

[r2] Betancourt AM Eltoum IA Desmond RA Russo J Lamartiniere CA 2010 *In utero* exposure to bisphenol A shifts the window of susceptibility for mammary carcinogenesis in the rat. Environ Health Perspect 118 1614 1619; 10.1289/ehp.1002148 20675265 PMC2974702

[r3] Biegel LB, Flaws JA, Hirshfield AN, O’Connor JC, Elliot GS, Ladics GS (1998). 90-day feeding and one-generation reproduction study in Crl:CD BR rats with 17β-estradiol.. Toxicol Sci.

[r4] Braun MM, Ahlbom A, Floderus B, Brinton LA, Hoover RN (1995). Effect of twinship on incidence of cancer of the testis, breast, and other sites (Sweden).. Cancer Causes Control.

[r5] Burridge E. 2008 Chemical profile: bisphenol A. ICIS Chem Business 274:48.

[r6] Calafat AM Kuklenyik Z Reidy JA Caudill SP Ekong J Needham JL 2005 Urinary concentrations of bisphenol A and 4-nonylphenol in a human reference population. Environ Health Perspect 113 391 395; 10.1289/ehp.7534 15811827 PMC1278476

[r7] Davis B, Fenton S. (2013). Mammary gland.

[r8] Doerge DR, Vanlandingham M, Twaddle NC, Delclos KB (2010). Lactational transfer of bisphenol A in Sprague-Dawley rats.. Toxicol Lett.

[r9] Durando M Kass L Piva J Sonnenschein C Soto AM Luque EH 2007 Prenatal bisphenol A exposure induces preneoplastic lesions in the mammary gland in Wistar rats. Environ Health Perspect 115 80 86; 10.1289/ehp.9282 17366824 PMC1797838

[r10] Ekbom A, Trichopoulos D, Adami HO, Hsieh CC, Lan SJ (1992). Evidence of prenatal influences on breast cancer risk.. Lancet.

[r11] Fernandez MF, Arrebola JP, Taoufiki J, Nafalón A, Ballesteros O, Pulgar R (2007). Bisphenol-A and chlorinated derivatives in adipose tissue of women.. Reprod Toxicol.

[r12] Herbst AL, Ulfelder H, Poskanzer DC (1971). Adenocarcinoma of the vagina: association of maternal stilbestrol therapy with tumor appearance in young women.. New Engl J Med.

[r13] Hoover RN, Hyer M, Pfeiffer RM, Adam E, Bond B, Cheville AL (2011). Adverse health outcomes in women exposed in utero to diethylstilbestrol.. N Engl J Med.

[r14] Ikezaki S, Takagi M, Tamura K (2011). Natural occurrence of neoplastic lesions in young Sprague-Dawley rats.. J Toxicol Pathol.

[r15] Ikezuki Y, Tsutsumi O, Takai Y, Kamei Y, Taketani Y (2002). Determination of bisphenol A concentrations in human biological fluids reveals significant early prenatal exposure.. Hum Reprod.

[r16] Institute of Laboratory Animal Resources, National Research Council. (2011). Guide for the Care and Use of Laboratory Animals. 8th ed. Washington, DC:National Academies Press.. http://www.nap.edu/catalog.php?record_id=12910.

[r17] Jenkins S Raghuraman N Eltoum I Carpenter M Russo J Lamartiniere CA 2009 Oral exposure to bisphenol A increases dimethylbenzanthracene-induced mammary cancer in rats. Environ Health Perspect 117 910 915; 10.1289/ehp.11751 19590682 PMC2702405

[r18] Lotz W, Krause R (1978). Correlation between the effects of neuroleptics on prolactin release, mammary stimulation and the vaginal cycle in rats.. J Endocrinol.

[r19] Lucas JN, Rudmann DG, Credille KM, Irizarry AR, Peter A, Snyder PW (2007). The rat mammary gland: morphologic changes as an indicator of systemic hormonal perturbations induced by xenobiotics.. Toxicol Pathol.

[r20] Maffini MV, Soto AM, Calabro JM, Ucci AA, Sonnenschein C (2004). The stroma as a crucial target in rat mammary gland carcinogenesis.. J Cell Sci.

[r21] Markey CM Luque EH, Munoz de Toromm, Sonnenschein C, Soto AM. 2001 In utero exposure to bisphenol A alters the development and tissue organization of the mouse mammary gland. Biol Reprod 65 1215 1223 11566746 10.1093/biolreprod/65.4.1215

[r22] Munoz de Toro MM, Markey CM, Wadia PR, Luque EH, Rubin BS, Sonnenschein C (2005). Perinatal exposure to bisphenol A alters peripubertal mammary gland development in mice.. Endocrinology.

[r23] Murray TJ, Maffini MV, Ucci AA, Sonnenschein C, Soto AM (2007). Induction of mammary gland ductal hyperplasias and carcinoma *in situ* following fetal bisphenol A exposure.. Reprod Toxicol.

[r24] Murray TJ, Ucci AA, Maffini MV, Sonnenschein C, Soto AM (2009). Histological analysis of low dose NMU effects in the rat mammary gland.. BMC Cancer.

[r25] National Toxicology Program. (2007). Toxicology and Carcinogenesis Study of Genistein (CAS No. 446-72-0) in Sprague-Dawley Rats (Feed Study). NTP TR 545. Research Triangle Park, NC:National Toxicology Program.. http://ntp.niehs.nih.gov/ntp/htdocs/LT_rpts/TR545.pdf.

[r26] National Toxicology Program. (2010). Toxicology and Carcinogenesis Study of Ethinyl Estradiol (CAS No. 57-63-6) in Sprague-Dawley Rats (Feed Study). NTP TR 548. Research Triangle Park, NC:National Toxicology Program.. http://ntp.niehs.nih.gov/ntp/htdocs/LT_rpts/TR548.pdf.

[r27] Potischman N, Troisi R (1999). *In utero* and early life exposures in relation to risk of breast cancer.. Cancer Causes Control.

[r28] Rubin BS, Lenkowski JR, Schaeberle CM, Vandenberg LN, Ronsheim PM, Soto AM (2006). Evidence of altered brain sexual differentiation in mice exposed perinatally to low environmentally relevant levels of bisphenol A.. Endocrinology.

[r29] Rubin BS, Murray MK, Damassa DA, King JC, Soto AM (2001). Perinatal exposure to low doses of bisphenol A affects body weight, patterns of estrous cyclicity, and plasma LH levels.. Environ Health Perspect.

[r30] Schönfelder G, Wittfoht W, Hopp H, Talsness CE, Paul M, Chahoud I (2002). Parent bisphenol A accumulation in the human maternal–fetal–placental unit.. Environ Health Perspect.

[r31] Son WC, Gopinath C (2004). Early occurrence of spontaneous tumors in CD-1 mice and Sprague-Dawley rats.. Toxicol Pathol.

[r32] Soto AM, Lin TM, Justicia H, Silvia RM, Sonnenschein C. (1992). An “in culture” bioassay to assess the estrogenicity of xenobiotics. In: Chemically Induced Alterations in Sexual Development: The Wildlife/Human Connection (Colborn T, Clement C, eds).

[r33] Soto AM, Sonnenschein C (2011). The tissue organization field theory of cancer: a testable replacement for the somatic mutation theory.. BioEssays.

[r34] Takahashi O, Oishi S (2000). Disposition of orally administered 2,2-bis(4-hydroxyphenyl) propane (bisphenol A) in pregnant rats and placental transfer to fetuses.. Environ Health Perspect.

[r35] Taylor JA vom Saal FS Welshons WV Drury B Rottinghaus G Hunt PA 2011 Similarity of bisphenol A pharmacokinetics in rhesus monkeys and mice: relevance for human exposure. Environ Health Perspect 119 422 430; 10.1289/ehp.1002514 20855240 PMC3080921

[r36] Tharp AP, Maffini MV, Hunt PA, Vandevoort CA, Sonnenschein C, Soto AM (2012). Bisphenol A alters the development of the rhesus monkey mammary gland.. Proc Natl Acad Sci USA.

[r37] Thomas P, Dong J (2006). Binding and activation of the seven-transmembrane estrogen receptor GPR30 by environmental estrogens: a potential novel mechanism of endocrine disruption.. J Steroid Biochem Mol Biol.

[r38] Troisi R, Hatch EE, Titus-Ernstoff L, Hyer M, Palmer JR, Robboy SJ (2007). Cancer risk in women prenatally exposed to diethylstilbestrol.. Int J Cancer.

[r39] U.S. EPA (U.S. Environmental Protection Agency). (1993). Bisphenol A. (CASRN 80-05-7).. http://www.epa.gov/iris/subst/0356.htm.

[r40] U.S. EPA (U.S. Environmental Protection Agency). (2005). Technology Transfer Network Air Toxics: 2005 National-Scale Air Toxics Assessment. Glossary of Key Terms.. http://www.epa.gov/ttn/atw/natamain/gloss1.html.

[r41] Vandenberg LN Chauhoud I Heindel JJ Padmanabhan V Paumgartten FJ Schoenfelder G 2010 Urinary, circulating and tissue biomonitoring studies indicate widespread exposure to bisphenol A. Environ Health Perspect 118 1055 1070; 10.1289/ehp.0901716 20338858 PMC2920080

[r42] Vandenberg LN, Colborn T, Hayes TB, Heindel JJ, Jacobs DR, Lee DH (2013). Regulatory decisions on endocrine disrupting chemicals should be based on the principles of endocrinology.. Reprod Toxicol.

[r43] Vandenberg LN, Hauser R, Marcus M, Olea N, Welshons WV (2007a). Human exposure to bisphenol A (BPA).. Reprod Toxicol.

[r44] Vandenberg LN, Maffini MV, Schaeberle CM, Ucci AA, Sonnenschein C, Rubin BS (2008). Perinatal exposure to the xenoestrogen bisphenol-A induces mammary intraductal hyperplasias in adult CD-1 mice.. Reprod Toxicol.

[r45] Vandenberg LN, Maffini MV, Wadia PR, Sonnenschein C, Rubin BS, Soto AM (2007b). Exposure to environmentally relevant doses of the xenoestrogen bisphenol-A alters development of the fetal mouse mammary gland.. Endocrinology.

[r46] vom Saal FS, Akingbemi BT, Belcher SM, Birnbaum LS, Crain DA, Eriksen M (2007). Chapel Hill bisphenol A expert panel consensus statement: integration of mechanisms, effects in animals and potential to impact human health at current levels of exposure.. Reprod Toxicol.

[r47] Wadia PR Cabaton NJ Borrero MD Rubin BS Sonnenschein C Shioda T 2013 Low-dose BPA exposure alters the mesenchymal and epithelial transcriptomes of the mouse fetal mammary gland. PLoS One 8 e63902; 10.1371/journal.pone.0063902 23704952 PMC3660582

[r48] Wadia PR Vandenberg LN Schaeberle CM Rubin BS Sonnenschein C Soto AM 2007 Perinatal bisphenol A exposure increases estrogen sensitivity of the mammary gland in diverse mouse strains. Environ Health Perspect 115 592 598; 10.1289/ehp.9640 17450229 PMC1852652

[r49] Watson CS, Bulayeva NN, Wozniak AL, Finnerty CC (2005). Signaling from the membrane via membrane estrogen receptor-α: estrogens, xenoestrogens, and phytoestrogens.. Steroids.

[r50] Weber Lozada K, Keri RA (2011). Bisphenol A increases mammary cancer risk in two distinct mouse models of breast cancer.. Biol Reprod.

[r51] Welshons WV Thayer KA Judy BM Taylor JA Curran EM vom Saal FS 2003 Large effects from small exposures. I. Mechanisms for endocrine-disrupting chemicals with estrogenic activity. Environ Health Perspect 111 994 1006; 10.1289/ehp.5494 12826473 PMC1241550

[r52] White SS, Calafat AM, Kuklenyik Z, Villanueva L, Zehr RD, Helfant L (2007). Gestational PFOA exposure of mice is associated with altered mammary gland development in dams and female offspring.. Toxicol Sci.

[r53] Ye X, Tao LJ, Needham LL, Calafat AM (2008). Automated on-line column-switching HPLC-MS/MS method for measuring environmental phenols and parabens in serum.. Talanta.

[r54] Yokota H, Iwano H, Endo M, Kobayashi T, Inoue H, Ikushiro S (1999). Glucuronidation of the environmental oestrogen bisphenol A by an isoform of UDP-glucuronosyltransferase, UGT2B1, in the rat liver.. Biochem J.

[r55] Zalko D, Jacques C, Duplan H, Bruel S, Perdu E (2011). Viable skin efficiently absorbs and metabolizes bisphenol A.. Chemosphere.

[r56] Zalko D Soto AM Dolo L Dorio C Ratahao E Debrauwer L 2003 Biotransformations of bisphenol A in a mammalian model: answers and new questions raised by low-dose metabolic fate studies in pregnant CD1 mice. Environ Health Perspect 111 309 319; 10.1289/ehp.5603 12611660 PMC1241388

