# Supplementary material for: Perinatally Administered Bisphenol A as a Potential Mammary Gland Carcinogen in Rats
Source: Environ Health Perspect. 2013 Jul 23;121(9):1040–6. doi: 10.1289/ehp.1306734 (PMC3764091; doi:10.1289/ehp.1306734)
Supplement: (430 KB) PDF [file ehp.1306734.s001.pdf]

Environmental Health Perspectives doi:10.1289/ehp.1306734

Supplemental Material

Perinatally Administered Bisphenol A Acts as a Mammary Gland Carcinogen in Rats

Nicole Acevedo, Barbara Davis, Cheryl M. Schaeberle, Carlos Sonnenschein, and Ana M. Soto

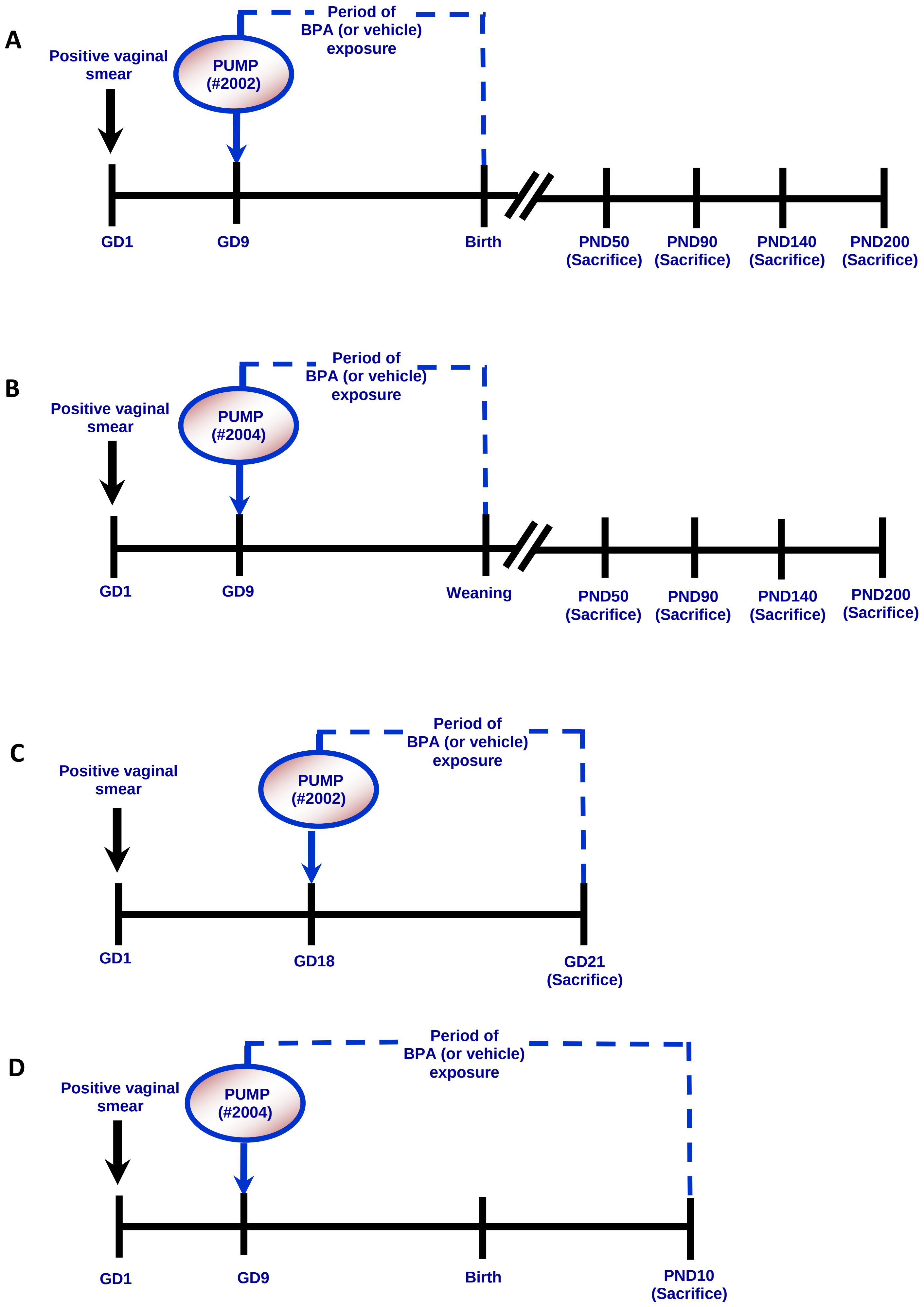
A
Positive vaginal smear
PUMP (#2002)
Period of BPA (or vehicle) exposure
GD1
GD9
Birth
PND50 (Sacrifice)
PND90 (Sacrifice)
PND140 (Sacrifice)
PND200 (Sacrifice)
B
Positive vaginal smear
PUMP (#2004)
Period of BPA (or vehicle) exposure
GD1
GD9
Weaning
PND50 (Sacrifice)
PND90 (Sacrifice)
PND140 (Sacrifice)
PND200 (Sacrifice)
C
Positive vaginal smear
PUMP (#2002)
Period of BPA (or vehicle) exposure
GD1
GD18
GD21 (Sacrifice)
D
Positive vaginal smear
PUMP (#2004)
Period of BPA (or vehicle) exposure
GD1
GD9
Birth
PND10 (Sacrifice)

Supplemental Material, Figure S1. (A) Experimental design for assessment of BPA exposure during gestation only. Dams (n=9-12) were implanted at GD9 with subcutaneous pumps designed to deliver continuously either vehicle or BPA (0.25, 2.5, 25, or 250 µg/kg/d). Mammary glands from female offspring were harvested at the time of sacrifice and assessed for development of lesions. (B) Experimental design for assessment of the effect of BPA exposure during gestation and lactation. Dams (n=9-12) were implanted at GD9 with subcutaneous pumps as previously described and mammary glands from female offspring were harvested at time of sacrifice and assessed for development of lesions. (C) Experimental design for measurement of serum BPA in dams and fetuses following exposure during gestation only. Dams (n=4-6) were implanted at G18 with subcutaneous pumps designed to deliver continuously either vehicle or 250 µg BPA/kg/d. Serum was collected from each dam and fetal sera from each litter (n=4-5) was pooled at time of sacrifice (GD21). (D) Experimental design for measurement of serum BPA in dams and pups following exposure during gestation and lactation. Dams (n=6) were implanted at GD9 with subcutaneous pumps designed to deliver continuously either vehicle or 250 µg BPA/kg/d. Serum was collected from each dam and pup sera from each litter (n=6) was pooled at time of sacrifice (PND10).
